# Aqua­{tris­[(1*H*-benzimidazol-2-yl-κ*N* ^3^)meth­yl]amine}­zinc 5-(dimethyl­amino)­naphthalene-1-sulfonate perchlorate 2.5-hydrate

**DOI:** 10.1107/S1600536811047453

**Published:** 2011-11-12

**Authors:** Zuo-an Xiao, Ting-ting Jiang

**Affiliations:** a School of Chemical Engineering and Food Science, Xiangfan University, Xiangfan 441053, People’s Republic of China

## Abstract

In the title compound, [Zn(C_24_H_21_N_7_)(H_2_O)](C_12_H_12_NO_3_S)(ClO_4_)·2.5H_2_O, the Zn^II^ ion is in a distorted trigonal–bipyramidal coordination geometry. In the crystal, N—H⋯O and O—H⋯O hydrogen bonds connect the components into a two-dimensional network parallel to (001). In addition, there are weak C—H⋯O hydrogen bonds.

## Related literature

For the biological and biochemical applications of benzimidazole compounds, see: Sundberg *et al.* (1977 ▶); Santoro *et al.* (2000 ▶). For the properties of tris­(1*H*-benzimidazol-2-ylmeth­yl)amine, see: Main (1992 ▶). For related structures, see: Tian *et al.* (2004 ▶); Wu *et al.* (2004 ▶); Li *et al.* (2005 ▶).
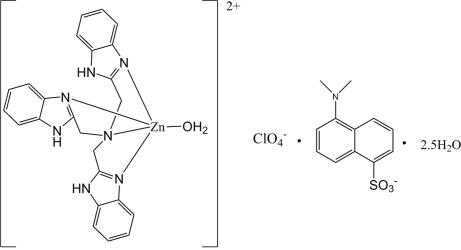


## Experimental

### 

#### Crystal data

[Zn(C_24_H_21_N_7_)(H_2_O)](C_12_H_12_NO_3_S)(ClO_4_)·2.5H_2_O
*M* *_r_* = 885.64
Monoclinic, 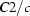

*a* = 26.327 (2) Å
*b* = 12.4462 (10) Å
*c* = 25.166 (2) Å
β = 100.242 (2)°
*V* = 8115.0 (11) Å^3^
*Z* = 8
Mo *K*α radiation
μ = 0.79 mm^−1^
*T* = 298 K
0.30 × 0.20 × 0.10 mm

#### Data collection

Bruker SMART CCD diffractometer
Absorption correction: multi-scan (*SADABS*; Sheldrick, 1996 ▶) *T*_min_ = 0.788, *T*_max_ = 0.925
32018 measured reflections
7146 independent reflections
5127 reflections with *I* > 2σ(*I*)
*R*_int_ = 0.079

#### Refinement

*R*[*F* ^2^ > 2σ(*F* ^2^)] = 0.062
*wR*(*F* ^2^) = 0.180
*S* = 0.99
7146 reflections
525 parameters
21 restraints
H-atom parameters constrained
Δρ_max_ = 0.96 e Å^−3^
Δρ_min_ = −0.51 e Å^−3^

### 

Data collection: *SMART* (Bruker, 2001 ▶); cell refinement: *SAINT* (Bruker, 2001 ▶); data reduction: *SAINT*; program(s) used to solve structure: *SHELXS97* (Sheldrick, 2008 ▶); program(s) used to refine structure: *SHELXL97* (Sheldrick, 2008 ▶); molecular graphics: *PLATON* (Spek, 2009) ▶; software used to prepare material for publication: *SHELXTL* (Sheldrick, 2008 ▶).

## Supplementary Material

Crystal structure: contains datablock(s) global, I. DOI: 10.1107/S1600536811047453/lh5361sup1.cif

Structure factors: contains datablock(s) I. DOI: 10.1107/S1600536811047453/lh5361Isup2.hkl

Additional supplementary materials:  crystallographic information; 3D view; checkCIF report

## Figures and Tables

**Table 1:** Hydrogen-bond geometry (Å, °)

*D*—H⋯*A*	*D*—H	H⋯*A*	*D*⋯*A*	*D*—H⋯*A*
O10—H10*B*⋯O6^i^	0.83	2.13	2.901 (10)	155
O10—H10*A*⋯O3^i^	0.83	1.93	2.756 (6)	175
N5—H5*A*⋯O9^ii^	0.86	2.19	2.938 (5)	146
N7—H7*A*⋯O4^iii^	0.86	2.12	2.914 (4)	153
N3—H3⋯O7^iii^	0.86	2.43	3.115 (9)	137
O9—H9*D*⋯O3	0.83	1.98	2.804 (5)	175
O9—H9*C*⋯O10	0.83	1.80	2.580 (7)	158
O1—H1*D*⋯O9	0.82	1.91	2.700 (4)	163
O1—H1*C*⋯O2	0.82	1.90	2.675 (4)	158
C13—H13⋯O6^i^	0.93	2.58	3.453 (9)	156
C17—H17*B*⋯O5^iii^	0.97	2.40	3.347 (7)	166

Symmetry codes: (i) 
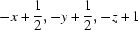
; (ii) 
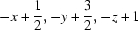
; (iii) 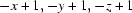.

**Fig. 1.**
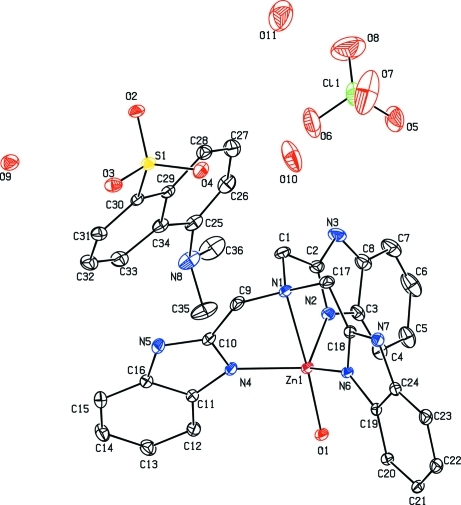
The molecular structure of (I), with displacement ellipsoids drawn at the 10% probability level. Hydrogen atoms are omitted for clarity.

**Fig. 2.**
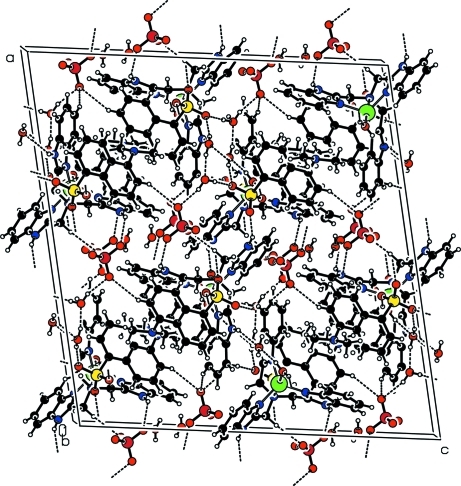
The crystal packing showing the hydrogen bonds shown as dashed lines.

## supplementary crystallographic information

### Comment

Imidazole (Im) and benzimidazole (Bzim) are common species in biological and biochemical structure and function (Sundberg *et al.*,1977; Santoro *et al.*, 2000). Tris(1*H*-benzimidazol-2-ylmethyl)-amine (NTB) is a benzimidazole-rich ligand, which has the advantage that the basicity of the coordinating group approximates to that of histidine (pKb: histidine = 7.96 and benzimidazole = 8.47; Main, 1992). Several examples of NTB-metal compounds have been reported (Tian *et al.*, 2004; Wu *et al.*, 2004; Li *et al.*, 2005), and the title compound, (I), is part of our effort in order to contribute to this research. Herein we report its crystal structure.

In (I) (Fig .1), the Zn^II^ ion is coordinated by four benzimidazole (bzim) N atoms of the NTB ligand and one O atom of H_2_O ligand, forming a five-coordinated distorted bipyramidal geometry. One amino N atom (N1) and one O atom (O1) of the H_2_O ligand occupy the axial positions, the other three bzim-N atoms (N2, N4 and N6) are located in the equatorial plane. All bond lengths and bond angles are as expected. In the crystal, N—H···O and O—H···O hydrogen bonds connect the components into a two-dimensional network parallel to (001). In addition there are weak intermolecular C—H···O hydrogen bonds (Fig. 2).

### Experimental

Zn(ClO_4_)_2_.6H_2_O (370 mg, 1 mmol) was dissolved in water (5 ml), dansyl acid (251 mg, 1 mmol) and NTB (407 mg, 1 mmol) were dissolved in ethanol (40 ml), then the two solutions were mixed and stirred at 333 k for 8 h. The pH of the mixture was maintained between 7–8 by addition of 1 mol.*L*^-1^ NaOH. The solution was filtered, yellow crystals suitable for X-ray diffraction studies were obtained after a week. Elemental analysis calculated: C 48.33, H 4.62, N 12.52%; found: C 48.66, H 4.49, N 12.84%.

### Refinement

All Hydrogen atoms were placed in calculated positions [*C*—*H*(methylene) = 0.97 Å, *N*—*H*(amine) = 0.86Å and C—H(aromatic) = 0.93 Å] and included in the refinement in a riding-motion approximation, with *U*_iso_(*H*)=1.5*U*_eq_(methyl C) and *U*_iso_(*H*)=1.2*U*_eq_(amine, methylene and aromatic C). Hydrogen atoms bonded to oxygen atoms were calculated and  placed at their indicated positions in the difference maps and refined with O-H=0.82-0.83Å and *U*_iso_(*H*)=1.5*U*_eq_(O). The half occupancy water molecule is close to a twofold rotation axis.

### Crystal data

**Table:**

[Zn(C_24_H_21_N_7_)(H_2_O)](C_12_H_12_NO_3_S)(ClO_4_)·2.5H_2_O	*F*(000) = 3672
*M**_r_* = 885.64	*D*_x_ = 1.450 Mg m^−^^3^
Monoclinic, *C*2/*c*	Mo *K*α radiation, λ = 0.71073 Å
Hall symbol: -C 2yc	Cell parameters from 7269 reflections
*a* = 26.327 (2) Å	θ = 2.4–23.2°
*b* = 12.4462 (10) Å	µ = 0.79 mm^−^^1^
*c* = 25.166 (2) Å	*T* = 298 K
β = 100.242 (2)°	Block, yellow
*V* = 8115.0 (11) Å^3^	0.30 × 0.20 × 0.10 mm
*Z* = 8	

### Data collection

**Table:**

Bruker SMART CCD diffractometer	7146 independent reflections
Radiation source: fine-focus sealed tube	5127 reflections with *I* > 2σ(*I*)
graphite	*R*_int_ = 0.079
φ and ω scans	θ_max_ = 25.0°, θ_min_ = 1.6°
Absorption correction: multi-scan (*SADABS*; Sheldrick, 1996)	*h* = −31→31
*T*_min_ = 0.788, *T*_max_ = 0.925	*k* = −14→14
32018 measured reflections	*l* = −29→29

### Refinement

**Table:**

Refinement on *F*^2^	Primary atom site location: structure-invariant direct methods
Least-squares matrix: full	Secondary atom site location: difference Fourier map
*R*[*F*^2^ > 2σ(*F*^2^)] = 0.062	Hydrogen site location: inferred from neighbouring sites
*wR*(*F*^2^) = 0.180	H-atom parameters constrained
*S* = 0.99	*w* = 1/[σ^2^(*F*_o_^2^) + (0.1174*P*)^2^] where *P* = (*F*_o_^2^ + 2*F*_c_^2^)/3
7146 reflections	(Δ/σ)_max_ = 0.001
525 parameters	Δρ_max_ = 0.96 e Å^−^^3^
21 restraints	Δρ_min_ = −0.51 e Å^−^^3^

### Special details

**Table:**

Geometry. All esds (except the esd in the dihedral angle between two l.s. planes) are estimated using the full covariance matrix. The cell esds are taken into account individually in the estimation of esds in distances, angles and torsion angles; correlations between esds in cell parameters are only used when they are defined by crystal symmetry. An approximate (isotropic) treatment of cell esds is used for estimating esds involving l.s. planes.
Refinement. Refinement of F^2^ against ALL reflections. The weighted R-factor wR and goodness of fit S are based on F^2^, conventional R-factors R are based on F, with F set to zero for negative F^2^. The threshold expression of F^2^ > 2sigma(F^2^) is used only for calculating R-factors(gt) etc. and is not relevant to the choice of reflections for refinement. R-factors based on F^2^ are statistically about twice as large as those based on F, and R- factors based on ALL data will be even larger.

### Fractional atomic coordinates and isotropic or equivalent isotropic displacement parameters (Å^2^)

**Table:**

	*x*	*y*	*z*	*U*_iso_*/*U*_eq_	Occ. (<1)
Zn1	0.368534 (15)	0.67112 (3)	0.425319 (17)	0.04460 (18)	
C1	0.40414 (18)	0.8930 (3)	0.3887 (2)	0.0747 (14)	
H1A	0.4331	0.9415	0.3893	0.090*	
H1B	0.3726	0.9338	0.3781	0.090*	
C2	0.40665 (16)	0.8048 (4)	0.3486 (2)	0.0657 (13)	
C3	0.39395 (15)	0.6491 (4)	0.30729 (18)	0.0640 (13)	
C4	0.38204 (17)	0.5451 (4)	0.29224 (18)	0.0680 (13)	
H4	0.3674	0.4991	0.3144	0.082*	
C5	0.3924 (2)	0.5108 (6)	0.2435 (2)	0.0934 (18)	
H5	0.3852	0.4406	0.2318	0.112*	
C6	0.4145 (3)	0.5854 (9)	0.2110 (3)	0.124 (3)	
H6	0.4207	0.5615	0.1777	0.149*	
C7	0.4267 (3)	0.6842 (7)	0.2244 (3)	0.103 (2)	
H7	0.4419	0.7295	0.2024	0.124*	
C8	0.41569 (18)	0.7176 (6)	0.2735 (2)	0.0799 (15)	
C9	0.37145 (17)	0.8994 (3)	0.4741 (2)	0.0653 (12)	
H9A	0.3721	0.9766	0.4688	0.078*	
H9B	0.3834	0.8847	0.5121	0.078*	
C10	0.31871 (16)	0.8594 (3)	0.45783 (18)	0.0577 (11)	
C11	0.25419 (15)	0.7558 (3)	0.42260 (16)	0.0498 (9)	
C12	0.22274 (16)	0.6785 (3)	0.39532 (19)	0.0618 (11)	
H12	0.2364	0.6171	0.3822	0.074*	
C13	0.16983 (18)	0.6951 (4)	0.3880 (2)	0.0768 (14)	
H13	0.1475	0.6439	0.3698	0.092*	
C14	0.14966 (19)	0.7880 (5)	0.4078 (2)	0.0857 (15)	
H14	0.1141	0.7973	0.4021	0.103*	
C15	0.18053 (19)	0.8651 (4)	0.4350 (2)	0.0770 (14)	
H15	0.1669	0.9263	0.4482	0.092*	
C16	0.23340 (17)	0.8479 (3)	0.44196 (19)	0.0593 (11)	
C17	0.45856 (16)	0.8302 (3)	0.4718 (2)	0.0640 (12)	
H17A	0.4686	0.8894	0.4966	0.077*	
H17B	0.4825	0.8269	0.4467	0.077*	
C18	0.45987 (14)	0.7271 (3)	0.50249 (16)	0.0478 (9)	
C19	0.44282 (13)	0.5649 (3)	0.52476 (14)	0.0407 (8)	
C20	0.42248 (15)	0.4624 (3)	0.52827 (16)	0.0468 (9)	
H20	0.3933	0.4392	0.5046	0.056*	
C21	0.44819 (15)	0.3965 (3)	0.56908 (16)	0.0535 (10)	
H21	0.4359	0.3274	0.5729	0.064*	
C22	0.49164 (16)	0.4313 (4)	0.60412 (18)	0.0627 (11)	
H22	0.5076	0.3845	0.6307	0.075*	
C23	0.51212 (16)	0.5323 (4)	0.60114 (18)	0.0594 (11)	
H23	0.5412	0.5552	0.6251	0.071*	
C24	0.48661 (13)	0.5985 (3)	0.55993 (15)	0.0459 (9)	
C25	0.2954 (2)	−0.0119 (4)	0.2644 (2)	0.0736 (14)	
C26	0.3384 (3)	0.0095 (4)	0.2441 (2)	0.0859 (16)	
H26	0.3434	−0.0244	0.2125	0.103*	
C27	0.3762 (2)	0.0826 (5)	0.2699 (2)	0.0878 (15)	
H27	0.4053	0.0962	0.2547	0.105*	
C28	0.37068 (18)	0.1332 (4)	0.31654 (19)	0.0664 (12)	
H28	0.3964	0.1787	0.3340	0.080*	
C29	0.32507 (15)	0.1158 (3)	0.33825 (16)	0.0526 (10)	
C30	0.31580 (15)	0.1641 (3)	0.38677 (17)	0.0504 (10)	
C31	0.26929 (18)	0.1555 (3)	0.4027 (2)	0.0655 (12)	
H31	0.2639	0.1882	0.4345	0.079*	
C32	0.22930 (18)	0.0971 (4)	0.3709 (2)	0.0803 (15)	
H32	0.1968	0.0953	0.3804	0.096*	
C33	0.23784 (18)	0.0439 (4)	0.3270 (2)	0.0744 (14)	
H33	0.2113	0.0037	0.3072	0.089*	
C34	0.28571 (18)	0.0474 (3)	0.31021 (17)	0.0591 (11)	
C35	0.2530 (3)	−0.1812 (5)	0.2739 (3)	0.128 (3)	
H35A	0.2784	−0.2337	0.2689	0.192*	
H35B	0.2191	−0.2115	0.2637	0.192*	
H35C	0.2582	−0.1601	0.3112	0.192*	
C36	0.2610 (3)	−0.1220 (6)	0.1863 (3)	0.142 (3)	
H36A	0.2666	−0.0605	0.1650	0.212*	
H36B	0.2294	−0.1567	0.1704	0.212*	
H36C	0.2892	−0.1713	0.1874	0.212*	
N1	0.40553 (13)	0.8473 (2)	0.44208 (15)	0.0571 (9)	
N2	0.38907 (12)	0.7073 (3)	0.35422 (14)	0.0554 (9)	
N3	0.42293 (16)	0.8138 (4)	0.30098 (19)	0.0860 (14)	
H3	0.4359	0.8708	0.2895	0.103*	
N4	0.30780 (12)	0.7648 (2)	0.43334 (13)	0.0518 (8)	
N5	0.27473 (13)	0.9102 (3)	0.46409 (15)	0.0638 (9)	
H5A	0.2730	0.9715	0.4794	0.077*	
N6	0.42641 (11)	0.6488 (2)	0.48872 (12)	0.0413 (7)	
N7	0.49602 (12)	0.7020 (3)	0.54441 (14)	0.0547 (8)	
H7A	0.5209	0.7428	0.5593	0.066*	
N8	0.25788 (19)	−0.0879 (3)	0.24062 (17)	0.0942 (15)	
O1	0.33343 (10)	0.52475 (19)	0.41288 (11)	0.0534 (7)	
H1C	0.3493	0.4753	0.4021	0.080*	
H1D	0.3093	0.5013	0.4258	0.080*	
O2	0.37520 (12)	0.3336 (2)	0.39794 (13)	0.0652 (8)	
O3	0.34350 (12)	0.2669 (2)	0.47625 (12)	0.0702 (8)	
O4	0.40978 (12)	0.1702 (2)	0.43981 (15)	0.0760 (9)	
Cl1	0.45345 (7)	0.03484 (16)	0.63565 (10)	0.1208 (6)	
O5	0.4653 (2)	0.1378 (4)	0.6203 (3)	0.163 (2)	
O6	0.4032 (2)	0.0348 (6)	0.6411 (3)	0.205 (3)	
O7	0.4865 (3)	0.0216 (7)	0.6843 (3)	0.296 (6)	
O8	0.4623 (4)	−0.0368 (7)	0.6007 (4)	0.289 (6)	
O9	0.26915 (15)	0.4297 (3)	0.47110 (14)	0.0938 (11)	
H9C	0.2412	0.3977	0.4653	0.141*	
H9D	0.2921	0.3842	0.4718	0.141*	
O10	0.1726 (2)	0.3805 (6)	0.4467 (3)	0.192 (3)	
H10A	0.1695	0.3348	0.4699	0.288*	
H10B	0.1445	0.3977	0.4279	0.288*	
S1	0.36503 (4)	0.23897 (8)	0.42816 (4)	0.0558 (3)	
O11	0.5051 (9)	0.2749 (12)	0.2823 (7)	0.254 (10)	0.50
H11A	0.5000	0.2489	0.2500	0.380*	
H11B	0.4937	0.2463	0.3071	0.380*	0.50

### Atomic displacement parameters (Å^2^)

**Table:**

	*U*^11^	*U*^22^	*U*^33^	*U*^12^	*U*^13^	*U*^23^
Zn1	0.0437 (3)	0.0331 (3)	0.0559 (3)	0.00009 (17)	0.0058 (2)	0.00499 (19)
C1	0.061 (3)	0.048 (3)	0.112 (4)	−0.004 (2)	0.005 (3)	0.033 (3)
C2	0.050 (2)	0.063 (3)	0.082 (3)	0.002 (2)	0.007 (2)	0.032 (3)
C3	0.041 (2)	0.099 (4)	0.051 (3)	0.010 (2)	0.006 (2)	0.023 (3)
C4	0.054 (3)	0.093 (4)	0.053 (3)	0.007 (2)	0.000 (2)	−0.001 (3)
C5	0.067 (3)	0.142 (5)	0.070 (4)	0.010 (3)	0.009 (3)	−0.010 (4)
C6	0.080 (4)	0.230 (10)	0.065 (4)	0.003 (6)	0.020 (3)	−0.001 (5)
C7	0.087 (4)	0.152 (7)	0.077 (4)	−0.010 (4)	0.032 (3)	0.021 (4)
C8	0.054 (3)	0.111 (5)	0.074 (4)	0.002 (3)	0.007 (3)	0.028 (3)
C9	0.069 (3)	0.031 (2)	0.092 (3)	−0.0013 (19)	0.004 (2)	−0.007 (2)
C10	0.065 (3)	0.034 (2)	0.075 (3)	0.0037 (19)	0.013 (2)	0.004 (2)
C11	0.049 (2)	0.045 (2)	0.056 (2)	0.0040 (17)	0.0124 (18)	0.0076 (18)
C12	0.053 (2)	0.054 (3)	0.078 (3)	0.0000 (19)	0.008 (2)	−0.004 (2)
C13	0.058 (3)	0.086 (4)	0.087 (4)	−0.008 (2)	0.012 (3)	0.000 (3)
C14	0.050 (3)	0.104 (4)	0.106 (4)	0.008 (3)	0.022 (3)	0.006 (4)
C15	0.065 (3)	0.069 (3)	0.103 (4)	0.015 (3)	0.031 (3)	−0.002 (3)
C16	0.060 (3)	0.049 (2)	0.073 (3)	0.0052 (19)	0.023 (2)	0.005 (2)
C17	0.050 (2)	0.045 (2)	0.094 (3)	−0.0103 (18)	0.004 (2)	0.009 (2)
C18	0.040 (2)	0.040 (2)	0.063 (3)	−0.0027 (16)	0.0072 (18)	−0.0046 (18)
C19	0.0393 (18)	0.0397 (19)	0.044 (2)	0.0057 (15)	0.0108 (16)	−0.0011 (16)
C20	0.047 (2)	0.041 (2)	0.053 (2)	−0.0013 (16)	0.0093 (18)	−0.0009 (17)
C21	0.059 (2)	0.044 (2)	0.059 (3)	0.0096 (18)	0.016 (2)	0.0112 (19)
C22	0.052 (2)	0.070 (3)	0.065 (3)	0.016 (2)	0.008 (2)	0.021 (2)
C23	0.044 (2)	0.069 (3)	0.061 (3)	0.0081 (19)	−0.0021 (19)	0.006 (2)
C24	0.0383 (19)	0.048 (2)	0.052 (2)	0.0010 (16)	0.0094 (17)	−0.0071 (18)
C25	0.095 (4)	0.050 (3)	0.067 (3)	0.000 (3)	−0.011 (3)	0.006 (2)
C26	0.117 (5)	0.070 (3)	0.067 (3)	0.007 (3)	0.008 (3)	−0.015 (3)
C27	0.094 (4)	0.085 (4)	0.088 (4)	0.001 (3)	0.028 (3)	−0.004 (3)
C28	0.070 (3)	0.053 (2)	0.075 (3)	−0.005 (2)	0.010 (2)	−0.005 (2)
C29	0.058 (2)	0.037 (2)	0.059 (3)	−0.0003 (17)	−0.002 (2)	0.0085 (18)
C30	0.050 (2)	0.034 (2)	0.064 (3)	0.0002 (16)	0.0020 (19)	0.0069 (18)
C31	0.066 (3)	0.059 (3)	0.070 (3)	−0.011 (2)	0.009 (2)	0.003 (2)
C32	0.053 (3)	0.087 (4)	0.095 (4)	−0.017 (3)	−0.001 (3)	0.010 (3)
C33	0.059 (3)	0.071 (3)	0.083 (4)	−0.019 (2)	−0.014 (3)	0.000 (3)
C34	0.069 (3)	0.046 (2)	0.054 (3)	−0.0046 (19)	−0.012 (2)	0.004 (2)
C35	0.177 (7)	0.070 (4)	0.115 (5)	−0.042 (4)	−0.035 (5)	0.012 (4)
C36	0.215 (9)	0.098 (5)	0.092 (5)	−0.017 (5)	−0.030 (5)	−0.035 (4)
N1	0.0526 (19)	0.0345 (17)	0.083 (3)	0.0009 (14)	0.0096 (18)	0.0141 (16)
N2	0.0480 (18)	0.058 (2)	0.060 (2)	0.0033 (15)	0.0080 (16)	0.0203 (17)
N3	0.072 (3)	0.103 (4)	0.086 (3)	−0.005 (2)	0.021 (2)	0.048 (3)
N4	0.0472 (18)	0.0351 (17)	0.072 (2)	0.0036 (14)	0.0066 (16)	0.0016 (15)
N5	0.067 (2)	0.0442 (19)	0.082 (3)	0.0065 (17)	0.018 (2)	−0.0101 (18)
N6	0.0400 (16)	0.0330 (15)	0.0501 (18)	−0.0022 (12)	0.0054 (14)	0.0021 (13)
N7	0.0394 (17)	0.0464 (19)	0.075 (2)	−0.0033 (14)	0.0022 (17)	−0.0067 (17)
N8	0.128 (4)	0.066 (3)	0.072 (3)	−0.015 (3)	−0.030 (3)	−0.010 (2)
O1	0.0514 (15)	0.0389 (14)	0.0686 (18)	−0.0043 (11)	0.0076 (13)	0.0018 (12)
O2	0.076 (2)	0.0407 (16)	0.080 (2)	−0.0132 (13)	0.0163 (17)	−0.0001 (14)
O3	0.087 (2)	0.0583 (19)	0.0651 (19)	−0.0154 (16)	0.0136 (17)	−0.0074 (15)
O4	0.0563 (17)	0.0536 (18)	0.106 (2)	−0.0028 (13)	−0.0171 (17)	−0.0031 (16)
Cl1	0.0852 (11)	0.1234 (15)	0.1551 (17)	−0.0241 (9)	0.0248 (12)	0.0337 (13)
O5	0.158 (5)	0.146 (4)	0.203 (6)	−0.020 (4)	0.081 (4)	0.062 (4)
O6	0.126 (5)	0.250 (8)	0.255 (7)	−0.067 (5)	0.081 (5)	0.008 (6)
O7	0.244 (9)	0.271 (10)	0.299 (10)	−0.114 (7)	−0.154 (8)	0.169 (8)
O8	0.322 (13)	0.240 (10)	0.294 (11)	0.017 (8)	0.025 (10)	−0.150 (9)
O9	0.123 (3)	0.064 (2)	0.105 (3)	−0.008 (2)	0.047 (2)	−0.0104 (19)
O10	0.106 (4)	0.253 (7)	0.229 (6)	0.007 (4)	0.060 (4)	0.135 (6)
S1	0.0561 (6)	0.0382 (5)	0.0695 (7)	−0.0089 (4)	0.0012 (5)	−0.0028 (5)
O11	0.217 (15)	0.153 (11)	0.39 (3)	0.016 (13)	0.04 (3)	−0.078 (12)

### Geometric parameters (Å, °)

**Table:**

Zn1—N2	2.010 (3)	C21—H21	0.9300
Zn1—N4	2.018 (3)	C22—C23	1.375 (6)
Zn1—N6	2.020 (3)	C22—H22	0.9300
Zn1—O1	2.042 (2)	C23—C24	1.399 (5)
Zn1—N1	2.406 (3)	C23—H23	0.9300
C1—N1	1.454 (6)	C24—N7	1.381 (5)
C1—C2	1.499 (7)	C25—C26	1.350 (8)
C1—H1A	0.9700	C25—N8	1.420 (6)
C1—H1B	0.9700	C25—C34	1.430 (7)
C2—N2	1.315 (5)	C26—C27	1.418 (8)
C2—N3	1.348 (6)	C26—H26	0.9300
C3—C4	1.369 (7)	C27—C28	1.363 (7)
C3—C8	1.397 (7)	C27—H27	0.9300
C3—N2	1.411 (6)	C28—C29	1.422 (6)
C4—C5	1.372 (7)	C28—H28	0.9300
C4—H4	0.9300	C29—C30	1.421 (6)
C5—C6	1.429 (10)	C29—C34	1.427 (6)
C5—H5	0.9300	C30—C31	1.358 (6)
C6—C7	1.300 (10)	C30—S1	1.775 (4)
C6—H6	0.9300	C31—C32	1.406 (6)
C7—C8	1.382 (8)	C31—H31	0.9300
C7—H7	0.9300	C32—C33	1.340 (7)
C8—N3	1.379 (7)	C32—H32	0.9300
C9—N1	1.460 (5)	C33—C34	1.400 (7)
C9—C10	1.463 (6)	C33—H33	0.9300
C9—H9A	0.9700	C35—N8	1.451 (7)
C9—H9B	0.9700	C35—H35A	0.9600
C10—N4	1.337 (5)	C35—H35B	0.9600
C10—N5	1.353 (5)	C35—H35C	0.9600
C11—C12	1.371 (6)	C36—N8	1.448 (7)
C11—N4	1.393 (5)	C36—H36A	0.9600
C11—C16	1.395 (5)	C36—H36B	0.9600
C12—C13	1.388 (6)	C36—H36C	0.9600
C12—H12	0.9300	N3—H3	0.8600
C13—C14	1.400 (7)	N5—H5A	0.8600
C13—H13	0.9300	N7—H7A	0.8600
C14—C15	1.361 (8)	O1—H1C	0.8168
C14—H14	0.9300	O1—H1D	0.8171
C15—C16	1.388 (6)	O2—S1	1.452 (3)
C15—H15	0.9300	O3—S1	1.467 (3)
C16—N5	1.371 (5)	O4—S1	1.444 (3)
C17—N1	1.478 (5)	Cl1—O8	1.303 (7)
C17—C18	1.494 (5)	Cl1—O6	1.353 (5)
C17—H17A	0.9700	Cl1—O7	1.380 (6)
C17—H17B	0.9700	Cl1—O5	1.390 (5)
C18—N6	1.319 (4)	O9—H9C	0.8265
C18—N7	1.327 (5)	O9—H9D	0.8267
C19—C24	1.387 (5)	O10—H10A	0.8292
C19—C20	1.393 (5)	O10—H10B	0.8329
C19—N6	1.399 (4)	O11—H11B	0.8200
C20—C21	1.392 (5)	O11—O11^i^	1.60 (3)
C20—H20	0.9300	O11—H11A	0.8634
C21—C22	1.384 (6)	O11—H11B	0.8200
			
N2—Zn1—N4	107.63 (13)	C26—C25—N8	122.7 (5)
N2—Zn1—N6	116.72 (12)	C26—C25—C34	119.0 (5)
N4—Zn1—N6	120.03 (12)	N8—C25—C34	118.3 (5)
N2—Zn1—O1	104.45 (13)	C25—C26—C27	121.5 (5)
N4—Zn1—O1	100.71 (11)	C25—C26—H26	119.3
N6—Zn1—O1	104.80 (11)	C27—C26—H26	119.3
N2—Zn1—N1	77.72 (14)	C28—C27—C26	121.1 (5)
N4—Zn1—N1	76.06 (12)	C28—C27—H27	119.4
N6—Zn1—N1	76.24 (11)	C26—C27—H27	119.4
O1—Zn1—N1	176.60 (11)	C27—C28—C29	119.2 (5)
N1—C1—C2	109.7 (3)	C27—C28—H28	120.4
N1—C1—H1A	109.7	C29—C28—H28	120.4
C2—C1—H1A	109.7	C30—C29—C28	123.5 (4)
N1—C1—H1B	109.7	C30—C29—C34	117.2 (4)
C2—C1—H1B	109.7	C28—C29—C34	119.3 (4)
H1A—C1—H1B	108.2	C31—C30—C29	121.4 (4)
N2—C2—N3	110.2 (5)	C31—C30—S1	118.1 (3)
N2—C2—C1	123.0 (4)	C29—C30—S1	120.5 (3)
N3—C2—C1	126.6 (4)	C30—C31—C32	119.7 (5)
C4—C3—C8	120.4 (5)	C30—C31—H31	120.1
C4—C3—N2	131.8 (4)	C32—C31—H31	120.1
C8—C3—N2	107.7 (5)	C33—C32—C31	120.4 (5)
C3—C4—C5	118.0 (5)	C33—C32—H32	119.8
C3—C4—H4	121.0	C31—C32—H32	119.8
C5—C4—H4	121.0	C32—C33—C34	121.7 (4)
C4—C5—C6	118.4 (7)	C32—C33—H33	119.1
C4—C5—H5	120.8	C34—C33—H33	119.1
C6—C5—H5	120.8	C33—C34—C29	118.9 (4)
C7—C6—C5	125.0 (7)	C33—C34—C25	121.8 (4)
C7—C6—H6	117.5	C29—C34—C25	119.3 (4)
C5—C6—H6	117.5	N8—C35—H35A	109.5
C6—C7—C8	115.8 (6)	N8—C35—H35B	109.5
C6—C7—H7	122.1	H35A—C35—H35B	109.5
C8—C7—H7	122.1	N8—C35—H35C	109.5
N3—C8—C7	132.6 (6)	H35A—C35—H35C	109.5
N3—C8—C3	105.0 (5)	H35B—C35—H35C	109.5
C7—C8—C3	122.4 (7)	N8—C36—H36A	109.5
N1—C9—C10	109.9 (3)	N8—C36—H36B	109.5
N1—C9—H9A	109.7	H36A—C36—H36B	109.5
C10—C9—H9A	109.7	N8—C36—H36C	109.5
N1—C9—H9B	109.7	H36A—C36—H36C	109.5
C10—C9—H9B	109.7	H36B—C36—H36C	109.5
H9A—C9—H9B	108.2	C1—N1—C9	114.8 (3)
N4—C10—N5	110.3 (4)	C1—N1—C17	113.0 (3)
N4—C10—C9	122.9 (4)	C9—N1—C17	113.7 (4)
N5—C10—C9	126.7 (4)	C1—N1—Zn1	104.6 (3)
C12—C11—N4	130.9 (4)	C9—N1—Zn1	103.4 (2)
C12—C11—C16	120.8 (4)	C17—N1—Zn1	105.9 (2)
N4—C11—C16	108.2 (3)	C2—N2—C3	107.4 (4)
C11—C12—C13	117.7 (4)	C2—N2—Zn1	117.2 (3)
C11—C12—H12	121.1	C3—N2—Zn1	135.1 (3)
C13—C12—H12	121.1	C2—N3—C8	109.7 (4)
C12—C13—C14	120.7 (5)	C2—N3—H3	125.1
C12—C13—H13	119.7	C8—N3—H3	125.1
C14—C13—H13	119.7	C10—N4—C11	106.7 (3)
C15—C14—C13	122.1 (5)	C10—N4—Zn1	116.5 (3)
C15—C14—H14	119.0	C11—N4—Zn1	136.6 (3)
C13—C14—H14	119.0	C10—N5—C16	108.8 (3)
C14—C15—C16	116.8 (5)	C10—N5—H5A	125.6
C14—C15—H15	121.6	C16—N5—H5A	125.6
C16—C15—H15	121.6	C18—N6—C19	105.4 (3)
N5—C16—C15	132.0 (4)	C18—N6—Zn1	118.7 (2)
N5—C16—C11	105.9 (3)	C19—N6—Zn1	135.8 (2)
C15—C16—C11	121.9 (4)	C18—N7—C24	107.8 (3)
N1—C17—C18	108.5 (3)	C18—N7—H7A	126.1
N1—C17—H17A	110.0	C24—N7—H7A	126.1
C18—C17—H17A	110.0	C25—N8—C36	116.1 (5)
N1—C17—H17B	110.0	C25—N8—C35	114.6 (4)
C18—C17—H17B	110.0	C36—N8—C35	109.6 (5)
H17A—C17—H17B	108.4	Zn1—O1—H1C	118.6
N6—C18—N7	112.7 (3)	Zn1—O1—H1D	128.4
N6—C18—C17	123.1 (4)	H1C—O1—H1D	110.0
N7—C18—C17	124.1 (3)	O8—Cl1—O6	111.4 (6)
C24—C19—C20	121.4 (3)	O8—Cl1—O7	110.9 (6)
C24—C19—N6	108.4 (3)	O6—Cl1—O7	112.5 (6)
C20—C19—N6	130.3 (3)	O8—Cl1—O5	111.5 (6)
C21—C20—C19	116.4 (4)	O6—Cl1—O5	107.5 (4)
C21—C20—H20	121.8	O7—Cl1—O5	102.7 (4)
C19—C20—H20	121.8	H9C—O9—H9D	107.4
C22—C21—C20	121.6 (4)	H10A—O10—H10B	112.9
C22—C21—H21	119.2	O4—S1—O2	111.5 (2)
C20—C21—H21	119.2	O4—S1—O3	113.2 (2)
C23—C22—C21	122.6 (4)	O2—S1—O3	111.96 (18)
C23—C22—H22	118.7	O4—S1—C30	107.01 (18)
C21—C22—H22	118.7	O2—S1—C30	107.56 (18)
C22—C23—C24	116.0 (4)	O3—S1—C30	105.05 (19)
C22—C23—H23	122.0	H11B—O11—O11^i^	139.0
C24—C23—H23	122.0	H11B—O11—H11A	122.5
N7—C24—C19	105.7 (3)	O11^i^—O11—H11B	139.0
N7—C24—C23	132.2 (4)	H11A—O11—H11B	122.5
C19—C24—C23	122.0 (4)		
			
N1—C1—C2—N2	28.6 (6)	N4—Zn1—N1—C9	28.2 (3)
N1—C1—C2—N3	−156.6 (4)	N6—Zn1—N1—C9	−98.0 (3)
C8—C3—C4—C5	0.4 (6)	N2—Zn1—N1—C17	−100.0 (3)
N2—C3—C4—C5	−178.8 (4)	N4—Zn1—N1—C17	148.1 (3)
C3—C4—C5—C6	−0.5 (7)	N6—Zn1—N1—C17	21.9 (3)
C4—C5—C6—C7	1.3 (10)	N3—C2—N2—C3	−0.2 (5)
C5—C6—C7—C8	−1.7 (11)	C1—C2—N2—C3	175.3 (4)
C6—C7—C8—N3	179.7 (6)	N3—C2—N2—Zn1	174.6 (3)
C6—C7—C8—C3	1.5 (9)	C1—C2—N2—Zn1	−9.9 (5)
C4—C3—C8—N3	−179.5 (4)	C4—C3—N2—C2	179.5 (5)
N2—C3—C8—N3	−0.1 (5)	C8—C3—N2—C2	0.2 (5)
C4—C3—C8—C7	−0.9 (7)	C4—C3—N2—Zn1	5.9 (7)
N2—C3—C8—C7	178.5 (5)	C8—C3—N2—Zn1	−173.3 (3)
N1—C9—C10—N4	22.6 (6)	N4—Zn1—N2—C2	64.8 (3)
N1—C9—C10—N5	−156.5 (4)	N6—Zn1—N2—C2	−73.6 (3)
N4—C11—C12—C13	−175.8 (4)	O1—Zn1—N2—C2	171.3 (3)
C16—C11—C12—C13	−0.3 (6)	N1—Zn1—N2—C2	−6.1 (3)
C11—C12—C13—C14	0.3 (7)	N4—Zn1—N2—C3	−122.1 (4)
C12—C13—C14—C15	−0.4 (9)	N6—Zn1—N2—C3	99.5 (4)
C13—C14—C15—C16	0.5 (8)	O1—Zn1—N2—C3	−15.7 (4)
C14—C15—C16—N5	175.7 (5)	N1—Zn1—N2—C3	167.0 (4)
C14—C15—C16—C11	−0.6 (7)	N2—C2—N3—C8	0.2 (5)
C12—C11—C16—N5	−176.6 (4)	C1—C2—N3—C8	−175.1 (4)
N4—C11—C16—N5	−0.2 (5)	C7—C8—N3—C2	−178.4 (6)
C12—C11—C16—C15	0.5 (7)	C3—C8—N3—C2	0.0 (5)
N4—C11—C16—C15	176.9 (4)	N5—C10—N4—C11	1.1 (5)
N1—C17—C18—N6	23.6 (6)	C9—C10—N4—C11	−178.1 (4)
N1—C17—C18—N7	−160.1 (4)	N5—C10—N4—Zn1	−175.5 (3)
C24—C19—C20—C21	0.5 (5)	C9—C10—N4—Zn1	5.3 (5)
N6—C19—C20—C21	179.7 (3)	C12—C11—N4—C10	175.4 (4)
C19—C20—C21—C22	−0.1 (6)	C16—C11—N4—C10	−0.5 (4)
C20—C21—C22—C23	0.1 (6)	C12—C11—N4—Zn1	−9.1 (7)
C21—C22—C23—C24	−0.6 (6)	C16—C11—N4—Zn1	175.0 (3)
C20—C19—C24—N7	179.1 (3)	N2—Zn1—N4—C10	−90.6 (3)
N6—C19—C24—N7	−0.2 (4)	N6—Zn1—N4—C10	46.2 (3)
C20—C19—C24—C23	−1.0 (5)	O1—Zn1—N4—C10	160.4 (3)
N6—C19—C24—C23	179.7 (3)	N1—Zn1—N4—C10	−18.5 (3)
C22—C23—C24—N7	−179.2 (4)	N2—Zn1—N4—C11	94.3 (4)
C22—C23—C24—C19	1.0 (6)	N6—Zn1—N4—C11	−129.0 (4)
N8—C25—C26—C27	−177.5 (5)	O1—Zn1—N4—C11	−14.8 (4)
C34—C25—C26—C27	5.5 (8)	N1—Zn1—N4—C11	166.3 (4)
C25—C26—C27—C28	0.6 (9)	N4—C10—N5—C16	−1.3 (5)
C26—C27—C28—C29	−2.8 (8)	C9—C10—N5—C16	177.9 (4)
C27—C28—C29—C30	179.5 (4)	C15—C16—N5—C10	−175.8 (5)
C27—C28—C29—C34	−1.3 (6)	C11—C16—N5—C10	0.9 (5)
C28—C29—C30—C31	172.0 (4)	N7—C18—N6—C19	−0.6 (4)
C34—C29—C30—C31	−7.2 (6)	C17—C18—N6—C19	176.0 (4)
C28—C29—C30—S1	−7.4 (5)	N7—C18—N6—Zn1	−179.6 (2)
C34—C29—C30—S1	173.4 (3)	C17—C18—N6—Zn1	−2.9 (5)
C29—C30—C31—C32	0.4 (6)	C24—C19—N6—C18	0.5 (4)
S1—C30—C31—C32	179.9 (3)	C20—C19—N6—C18	−178.7 (4)
C30—C31—C32—C33	4.4 (7)	C24—C19—N6—Zn1	179.2 (3)
C31—C32—C33—C34	−2.2 (8)	C20—C19—N6—Zn1	−0.1 (6)
C32—C33—C34—C29	−4.8 (7)	N2—Zn1—N6—C18	57.5 (3)
C32—C33—C34—C25	178.0 (4)	N4—Zn1—N6—C18	−75.5 (3)
C30—C29—C34—C33	9.2 (6)	O1—Zn1—N6—C18	172.5 (3)
C28—C29—C34—C33	−170.0 (4)	N1—Zn1—N6—C18	−10.9 (3)
C30—C29—C34—C25	−173.5 (4)	N2—Zn1—N6—C19	−121.0 (3)
C28—C29—C34—C25	7.3 (6)	N4—Zn1—N6—C19	106.0 (3)
C26—C25—C34—C33	167.9 (5)	O1—Zn1—N6—C19	−6.0 (4)
N8—C25—C34—C33	−9.3 (6)	N1—Zn1—N6—C19	170.6 (3)
C26—C25—C34—C29	−9.4 (7)	N6—C18—N7—C24	0.5 (4)
N8—C25—C34—C29	173.4 (4)	C17—C18—N7—C24	−176.1 (4)
C2—C1—N1—C9	−140.5 (4)	C19—C24—N7—C18	−0.2 (4)
C2—C1—N1—C17	86.9 (4)	C23—C24—N7—C18	180.0 (4)
C2—C1—N1—Zn1	−27.9 (4)	C26—C25—N8—C36	−14.9 (8)
C10—C9—N1—C1	80.9 (4)	C34—C25—N8—C36	162.1 (5)
C10—C9—N1—C17	−146.7 (3)	C26—C25—N8—C35	114.6 (7)
C10—C9—N1—Zn1	−32.4 (4)	C34—C25—N8—C35	−68.4 (7)
C18—C17—N1—C1	−141.5 (4)	C31—C30—S1—O4	126.5 (3)
C18—C17—N1—C9	85.3 (4)	C29—C30—S1—O4	−54.1 (4)
C18—C17—N1—Zn1	−27.6 (4)	C31—C30—S1—O2	−113.5 (3)
N2—Zn1—N1—C1	19.7 (3)	C29—C30—S1—O2	65.9 (3)
N4—Zn1—N1—C1	−92.2 (3)	C31—C30—S1—O3	5.9 (4)
N6—Zn1—N1—C1	141.5 (3)	C29—C30—S1—O3	−174.7 (3)
N2—Zn1—N1—C9	140.2 (3)		

Symmetry codes: (i) −*x*+1, *y*, −*z*+1/2.

### Hydrogen-bond geometry (Å, °)

**Table:**

*D*—H···*A*	*D*—H	H···*A*	*D*···*A*	*D*—H···*A*
O10—H10B···O6^ii^	0.83	2.13	2.901 (10)	155.
O10—H10A···O3^ii^	0.83	1.93	2.756 (6)	175.
N5—H5A···O9^iii^	0.86	2.19	2.938 (5)	146.
N7—H7A···O4^iv^	0.86	2.12	2.914 (4)	153.
N3—H3···O7^iv^	0.86	2.43	3.115 (9)	137.
O9—H9D···O3	0.83	1.98	2.804 (5)	175.
O9—H9C···O10	0.83	1.80	2.580 (7)	158.
O1—H1D···O9	0.82	1.91	2.700 (4)	163.
O1—H1C···O2	0.82	1.90	2.675 (4)	158.
C13—H13···O6^ii^	0.93	2.58	3.453 (9)	156.
C17—H17B···O5^iv^	0.97	2.40	3.347 (7)	166.

Symmetry codes: (ii) −*x*+1/2, −*y*+1/2, −*z*+1; (iii) −*x*+1/2, −*y*+3/2, −*z*+1; (iv) −*x*+1, −*y*+1, −*z*+1.
